# Androgen receptor suppresses β-adrenoceptor-mediated CREB activation and thermogenesis in brown adipose tissue of male mice

**DOI:** 10.1016/j.jbc.2022.102619

**Published:** 2022-10-19

**Authors:** Naoki Harada, Keitaro Kubo, Teruaki Onishi, Tomoya Kitakaze, Tsuyoshi Goto, Hiroshi Inui, Ryoichi Yamaji

**Affiliations:** 1 Division of Applied Life Sciences, Graduate School of Life and Environmental Sciences, Osaka Prefecture University, Sakai, Osaka, Japan; 2 Department of Applied Biological Chemistry, Graduate School of Agriculture, Osaka Metropolitan University, Sakai, Osaka, Japan; 3 Division of Food Science and Biotechnology, Graduate School of Agriculture, Kyoto University, Uji, Kyoto, Japan; 4 Department of Health and Nutrition, Otemae University, Osaka, Japan

**Keywords:** adipose tissue metabolism, adrenergic receptor, androgen receptor, cAMP response element-binding protein, core body temperature, gene knockout, norepinephrine, uncoupling protein, sex differences, testosterone, AR, androgen receptor, ARE, androgen response element, ARKO, androgen receptor knockout, BAT, brown adipose tissue, β3-AdR, β3-adrenoceptor, CRE, cAMP response element, CREB, cAMP-response element-binding protein, DHT, dihydrotestosterone, iWAT, subcutaneous inguinal white adipose tissue, NTD, N-terminal domain, UCP1, uncoupling protein 1

## Abstract

Thermoregulation is a process by which core body temperature is maintained in mammals. Males typically have a lower body temperature than females. However, the effects of androgens, which show higher levels in males, on adrenergic receptor-mediated thermogenesis remain unclear. Here, we demonstrate that androgen–androgen receptor (AR) signaling suppresses the β-adrenergic agonist-induced rise of core body temperature using castrated and AR knockout (ARKO) male mice. Furthermore, *in vitro* mechanistic studies show that activated AR inhibits cAMP response element (CRE)-mediated transcription by suppressing cAMP response element-binding protein (CREB) phosphorylation. The elevation of body temperature induced by the β-adrenergic agonist CL316243 was higher in ARKO and castrated mice than in the control mice. Similarly, CL316243 induced a greater increase in *Uncoupling protein 1* (*Ucp1*) expression and CREB phosphorylation in the brown adipose tissue of ARKO mice than in that of controls. We determined that activation of AR by dihydrotestosterone suppressed β3-agonist- or forskolin-induced CRE-mediated transcription, which was prevented by AR antagonist. AR activation also suppressed CREB phosphorylation induced by forskolin. Moreover, we found AR nuclear localization, but not transcriptional activity, was necessary for the suppression of CRE-mediated transcription. Finally, modified mammalian two-hybrid and immunoprecipitation analyses suggest nuclear AR and CREB form a protein complex both in the presence and absence of dihydrotestosterone and forskolin. These results suggest androgen–AR signaling suppresses β-adrenoceptor-induced UCP1-mediated brown adipose tissue thermogenesis by suppressing CREB phosphorylation, presumably owing to a protein complex with AR and CREB. This mechanism explains sexual differences in body temperature, at least partially.

Core body temperature is mainly regulated by the autonomic nervous system. Vasoconstriction regulates core body temperature by controlling heat loss through the skin, whereas shivering and nonshivering thermogenesis contribute heat production. Thus, while thermoregulation is extremely complex (1), brown adipose tissue (BAT) plays a key role in nonshivering thermogenesis (2). This metabolic process involves the actions of uncoupling protein 1 (UCP1), an inner mitochondrial membrane protein with proton transport activity that facilitates the production of heat instead of ATP (2, 3, 4). It was previously thought that BAT is only present and metabolically active in infants and regressed in the early years of life in humans. However, studies using PET/CT show that BAT is present in the paracervical and supraclavicular region of human adults (5, 6, 7). In BAT, UCP1 increases in response to noradrenaline–adrenergic receptor-mediated activation of the cAMP/PKA/cAMP response elementbinding protein (CREB) and p38MAPK pathway (8). β3-adrenoceptor (β3-AdR) is the most abundant and crucial adrenoceptor for UCP1 induction in BAT (3). Cold exposure is known to induce noradrenaline release from sympathetic nerves and stimulates BAT both in rodents and humans (2), indicating the role of BAT thermogenesis under stressed conditions. Recent studies have shown that thermoneutral conditions (approximately 30 °C in mice) result in BAT whitening and the loss of BAT thermogenic capacity (9). In addition, diet-induced adrenaline-mediated thermogenesis is abolished in UCP-1 ablated mice (10). Collectively, it is reasonable to assume that UCP1-dependent BAT thermogenesis is functional not only under severely stressed conditions (*i.e.*, cold exposure) but also under normal homeostatic conditions below the thermoneutral temperature for breeding mice.

Androgen receptor (AR) is a member of the nuclear receptor superfamily of ligand-activated transcription factors. Androgens such as testosterone and dihydrotestosterone (DHT) activate AR as a ligand. An increase of testosterone at puberty initiates sexual maturation in males, which contributes to the formation of sex differences in vertebrates (11, 12, 13, 14). There is a known sexual difference in core body temperature, with males having a lower core body temperature than females, both in mice (15) and humans (16, 17). Several studies have reported that BAT activity detected by PET/CT imaging is lower in men than in women (18), and UCP1 levels are also lower in the BAT of male mice than in that of female mice (18, 19). While estrogens modulate body temperature both in rodents and humans (20, 21), androgen ablation by AR knockout (ARKO) in males, but not in females, and male castration after puberty both increase body temperature in mice (22, 23, 24, 25). This suggests that male androgen signaling may also contribute to sexual differences in body temperature. This notion is supported by the finding that the activation of AR suppresses *Ucp1* expression in primary brown adipocytes from male mice (26). Collectively, the function of androgens in BAT might explain sexual differences in the body temperature (15, 16, 17). However, it has not been elucidated whether androgens could affect β-agonist-induced increases in thermogenesis and body temperature *in vivo*.

Crosstalk between PKA signaling and AR has been reported, such as PKA signaling enhancing the transcriptional activity of AR (27). However, the effect of androgens on PKA signaling has not yet been clarified. In this study, we examined the effects of androgen–AR signaling in adrenoceptor-mediated BAT thermogenesis *in vivo* and dissected the molecular mechanisms underlying the effect of androgens on thermogenesis *in vitro*.

## Results

### Core body temperature was higher in castrated and ARKO male mice both before and after the administration of β3-AdR agonist

**Figure 1:**
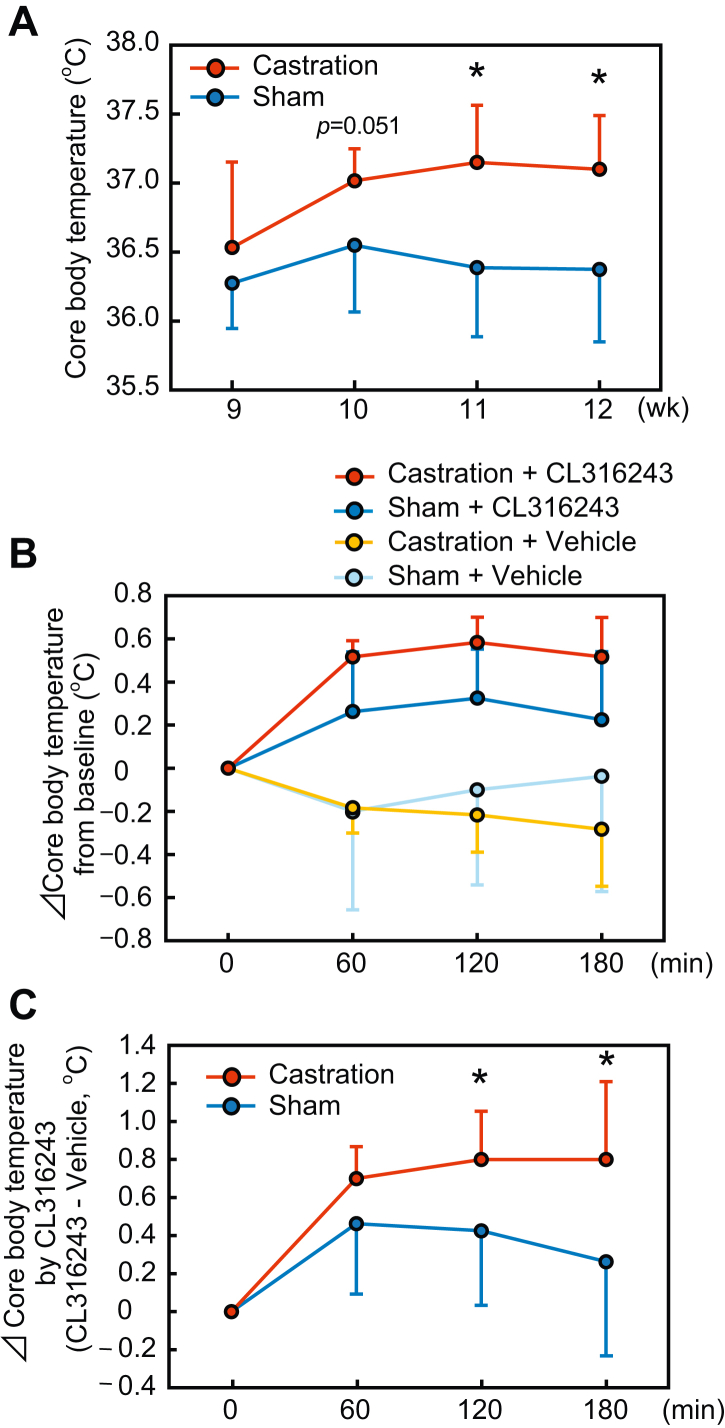
**Core body temperature was elevated in castrated male mice both before and after administration of β3-agonist.** Rectal body temperature was measured in castrated and sham-operated control male mice operated at 8-weeks of age. *A*, core body temperature of castrated mice during the study period. *B*, core body temperature changes after administration of the β3-adrenoceptor ligand CL316243 or vehicle in sham-operated or castrated mice from time 0. *C*, difference in core body temperature changes after administration of CL316243 or vehicle in each mouse. Data are expressed as means ± SD. (sham: n = 8; castration: n = 6). *Asterisks* indicate statistically significant differences between sham-operated and castrated mice at the same time point (*p* < 0.05).

Male mice castrated at 8 weeks of age showed an increase in core body temperature from the second week after the surgery (Fig. 1*A*). To evaluate the effect of androgen signaling on heat production, changes in body temperature after intraperitoneal injection of CL316243, a highly specific β3-AdR agonist (28), were determined (Fig. 1*B*). Both saline and CL316243 were administered to individual mice for two consecutive days. Subsequently, the change in body temperature in each mouse was calculated by subtracting the body temperature changes during the saline treatment period from those during the CL316243 treatment period. As shown in Figure 1*C*, treatment with CL316243 raised the body temperature of castrated mice more than that of their corresponding controls.

**Figure 2:**
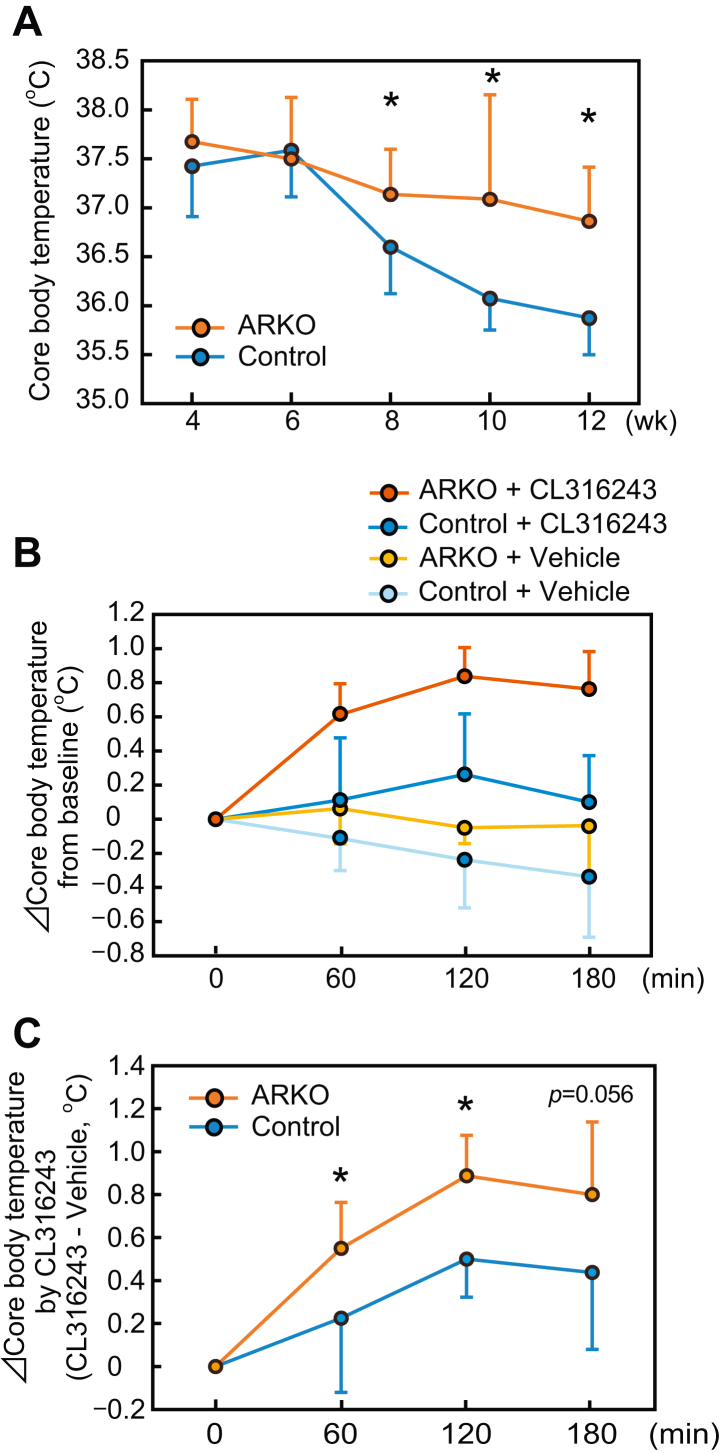
**Core body temperature was elevated in ARKO male mice both before and after administration of β3-agonist.***A*, core body temperature of ARKO or control mice during the study period (4–12 weeks old). *B*, core body temperature changes after administration of the β3-adrenoceptor ligand CL316243 or vehicle in ARKO or control mice from time 0. *C*, difference in core body temperature changes after the administration of CL316243 and vehicle in each mouse. Data are expressed as means ± SD. (control: n = 8; ARKO: n = 8). *Asterisks* indicate statistically significant differences between control and ARKO mice at the same time point (*p* < 0.05). ARKO, androgen receptor knockout.

To determine the involvement of AR in androgen-suppressed body temperature, ARKO mice were used. Analyses from the weaning period show that the core body temperature of ARKO mice was higher than that of the control mice of the same litter in an age-dependent manner (Fig. 2*A*). This difference in body temperature became apparent at 8 weeks of age. Intraperitoneal injection of CL316243 elicited a greater increase in the body temperature of ARKO mice than in that of control mice (Fig. 2, *B* and *C*). These results indicate that androgen–AR signaling suppresses adrenoceptor-mediated heat production in male mice.

### β3-AdR agonist upregulated Ucp1 expression in the BAT of ARKO mice to a greater extent than in the BAT of control mice

**Figure 3:**
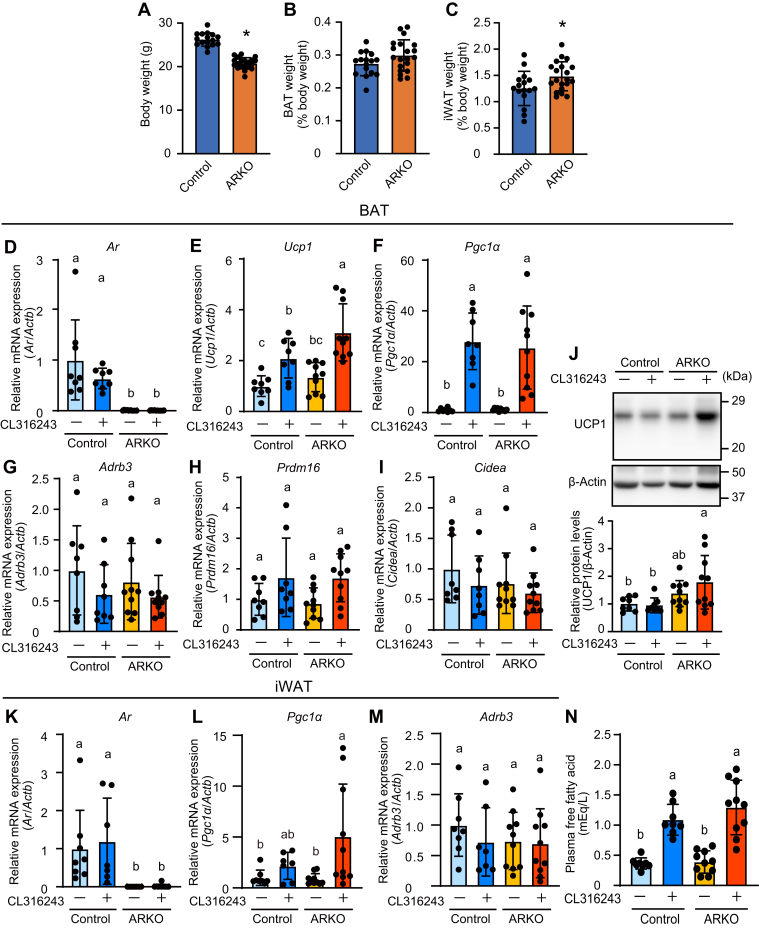
***Ucp1* expression in BAT was upregulated in ARKO mice to a greater extent than in control mice during β3-**adrenoceptor **agonist treatment.***A*, body weight, *B*, BAT weight, and *C*, iWAT weight of ARKO mice. Data are expressed as means ± SD. (control: n = 16; ARKO: n = 20). Expression of (*D*) *Ar*, (*E*) *Ucp1*, (*F*) *Pgc1α*, (*G*) *Adrb3*, (*H*) *Prdm16*, (*I*) *Cidea* mRNAs, and (*J*) UCP1 protein in BAT of ARKO mice 2 h after administration of vehicle or the β3-adrenoceptor agonist CL316243. Expressions of (*K*) *Ar*, (*L*) *Pgc1α*, and (*M*) *Adrb3* in iWAT of ARKO mice. *N*, plasma-free fatty acid levels. Data are expressed as the means ± SD (control + vehicle: n = 8; control + CL316243: n = 8; ARKO + vehicle: n = 10; ARKO + CL316243: n = 10). Asterisks and different letters indicate statistically significant differences (*p* < 0.05). ARKO, androgen receptor knockout; BAT, brown adipose tissue; iWAT, subcutaneous inguinal white adipose tissue.

ARKO caused a decrease, no effect, and an increase in body weight, BAT weight, and subcutaneous inguinal white adipose tissue (iWAT) weight, respectively (Fig. 3, *A*–*C*). To delineate the mechanism underlying the effect of AR-deficiency on CL316243-mediated increases in body temperature, gene expression profiling in BAT was conducted. *Ar* expression was eliminated in ARKO mice (Fig. 3*D*). *Ucp1* expression was induced by CL316243 to a greater extent in ARKO mice than in control mice (Fig. 3*E*). PGC1α was induced by β3-AdR stimuli and contributed to the induction of UCP1 (8), whereas there was no difference in the level of *Pgc1α* expression induced by the β3-AdR agonist between ARKO and control mice (Fig. 3*F*). The expression of *β3-AdR* and BAT marker genes (*Prdm16* and *Cidea*) was not significantly affected by CL316243 treatment (Fig. 3, *G*–*I*). In addition, the basal expression of these BAT genes also did not differ between ARKO and control mice, suggesting that AR does not affect the differentiation of BAT. UCP1 protein levels were higher in ARKO mice than in control mice when CL316243 was administered, whereas they did not differ between the control and ARKO mice when CL316243 was not administered (Fig. 3*J*), likely reflecting mRNA levels.

We also examined the effects of ARKO on CL316243 response in iWAT. *Ar* expression was ablated in ARKO mice (Fig. 3*K*). *Ucp1* expression was extremely low in iWAT (data not shown). The expression of UCP1 in iWAT is induced by intermittent β-AdR stimulation (*e.g.*, cold exposure or subsequent β-agonist treatment) accompanying the browning of the iWAT. In our study, it is assumed that a single administration of the β-agonist CL316243 cannot induce UCP1 expression in iWAT. Unlike in BAT, the β3-AdR agonist-induced *Pgc1α* expression was higher in ARKO mice than in control mice (Fig. 3*L*). This inconsistency could be caused by differences in the regulation of *Pgc1α* by PKA *via* ATF2 (8) in BAT and *via* CREB in WAT (29). The expression of *β3-AdR* was not significantly affected by ARKO and CL316243 treatment (Fig. 3*M*). After β3-AdR stimuli, free fatty acids are produced by the lipolysis of triglycerides and released from WAT. They are utilized in BAT as a fuel for thermogenesis (30). Thus, plasma-free fatty acid levels could reflect the β3-AdR signaling in WAT and may influence on the heat production in BAT. Free fatty acid levels in the plasma were increased by CL316243 in both control and ARKO groups, but the extents of induction did not differ between the two groups (Fig. 3*N*).

### β3-agonist treatment induced phosphorylation of CREB in the BAT of ARKO mice more effectively than in the BAT of control mice

**Figure 4:**
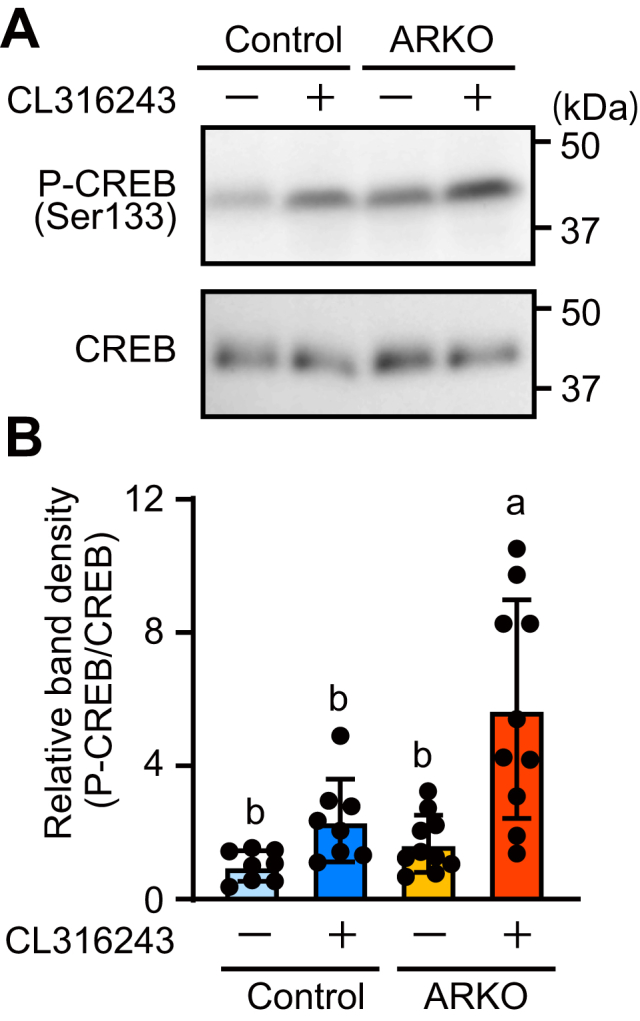
**Level of CREB phosphorylation inbrown adipose tissuewas increased in ARKO mice to a greater extent than in control mice during β3-adrenoceptor agonist treatment**. *A*, Western blotting of phosphorylated (Ser133) or total CREB levels 2 h after administration of vehicle or CL316243 in BAT of ARKO and control mice. *B*, the band intensity of P-CREB was expressed relative to the band intensity of total CREB. Data are expressed as means ± SD. (control + vehicle: n = 8; control + CL316243: n = 8; ARKO + vehicle: n = 10; ARKO + CL316243: n = 10). Different letters indicate statistically significant differences (*p* < 0.05). ARKO, androgen receptor knockout; CREB, cAMP-response element-binding protein

We determined the effects of ARKO on β-adrenoceptor-activated PKA signaling by measuring the levels of CREB phosphorylation. In multiple comparisons, CL316243 treatment had no significant effect on CREB phosphorylation in the control mice but led to a significant increase in the ARKO mice; there was a significant difference between the two groups (Fig. 4, *A* and *B*). There was a significant difference (*p* < 0.05, Student’s *t* test) in the direct comparison between the treated and untreated control mice. These *in vivo* studies suggest that AR signaling suppresses β3-agonist-induced *Ucp1* expression by suppressing CREB phosphorylation in BAT.

### Activated AR repressed the PKA/CREB/CRE pathway *in vitro*

**Figure 5:**
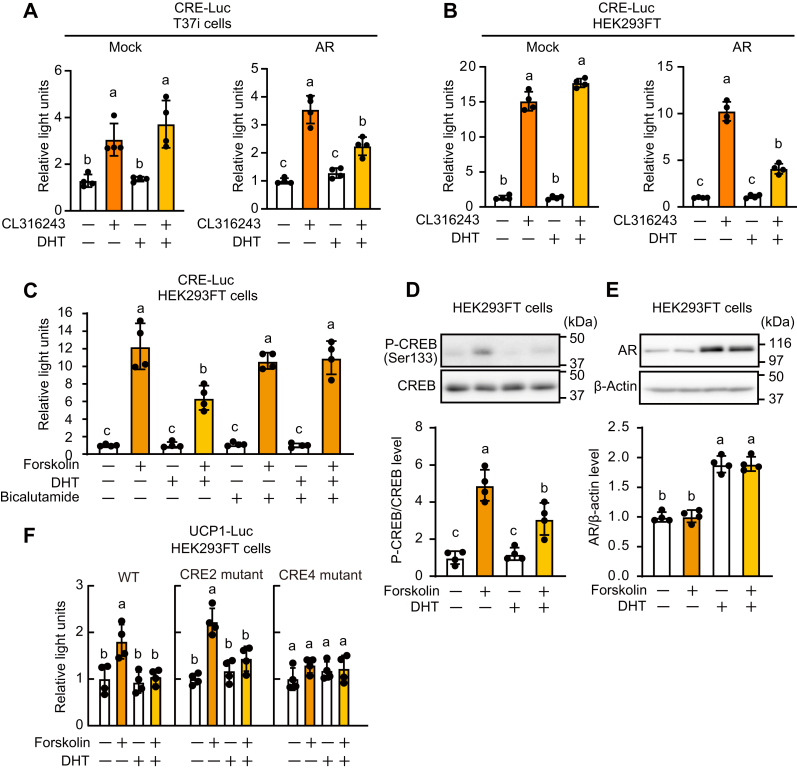
**Activation of AR suppressed β3-A-stimulatedCRE-mediated gene expression *in vitro*.***A*–*D* and *F*, T37i or HEK293FT cells were transfected with β3-adrenoceptor expression vector (pcDNA3.2-β3-AdR-V5), AR expression vector (pcDNA-3.1 (mock) or pcDNA3.1-AR), luciferase reporter vector (p4xCRE-TATA-Luc2P or WT or mutant pUCP1-pro-Luc2P), and *Renilla* luciferase reporter vector (pGL4.74[*hRluc*/TK]) for 24 h. Cells were incubated in the presence of 10 nM DHT for 16 h and then stimulated with 10 μM CL316243 or 10 μM forskolin for an additional 4 h (*A*–*C* and *F*) or 5 min (*D* and *E*). *A*–*C* and *F*, luciferase reporter assay was performed. *D*, Western blotting with anti-pCREB (Ser133) and anti-CREB was performed, and band intensity was calculated. *E*, Western blotting with anti-AR and anti-β-actin was performed, and band intensity was calculated. Data are expressed as means ± SD. (n = 4). Different letters or *asterisks* indicate significant differences (*p* < 0.05). AR, androgen receptor; CRE, cAMP response element; CREB, cAMP-response element-binding protein; UCP1, uncoupling protein 1; DHT, dihydrotestosterone.

We next examined the mechanism by which AR suppresses CRE activation *in vitro* with overexpression experiments. In T37i brown adipocytes, CL316243 activated CRE-mediated transcriptional activity, which was attenuated in the presence of DHT when cells exogenously expressed AR (Fig. 5*A*). Since similar results were observed with HEK293FT cells (Fig. 5*B*), subsequent experiments were performed with HEK293FT cells. AR activation also suppressed forskolin-induced CRE activity, which was abrogated by bicalutamide, an AR antagonist (Fig. 5*C*). Similarly, DHT inhibited the phosphorylation of CREB at Ser133, which was induced by forskolin (Fig. 5*D*). AR levels were increased by DHT but not affected by forskolin (Fig. 5*E*). *Ucp1* promoter activity was also induced by forskolin, which was attenuated by DHT in AR-overexpressed HEK293FT cells (Fig. 5*F*, left panel). As CREB regulates *Ucp1* expression *via* the CRE4 site, but not the CRE2 site, of the *Ucp1* promoter (8), we constructed *Ucp1* promoters mutated at the CRE2 or CRE4 sites. Forskolin induced the *Ucp1* promoter activity of the CRE2 mutant but failed to induce the activity of the CRE4 mutant (Fig. 5*F*, middle and right panels). AR suppressed the promoter activity of the CRE2 mutant but did not affect the activity of the CRE4 (CREB binding site) mutant. Collectively, these results suggest that agonist-activated AR inhibits CRE4-mediated *Ucp1* gene expression by suppressing the activation of CREB.

### Nuclear AR suppressed CRE-mediated gene expression independent of AR transcriptional activity

**Figure 6:**
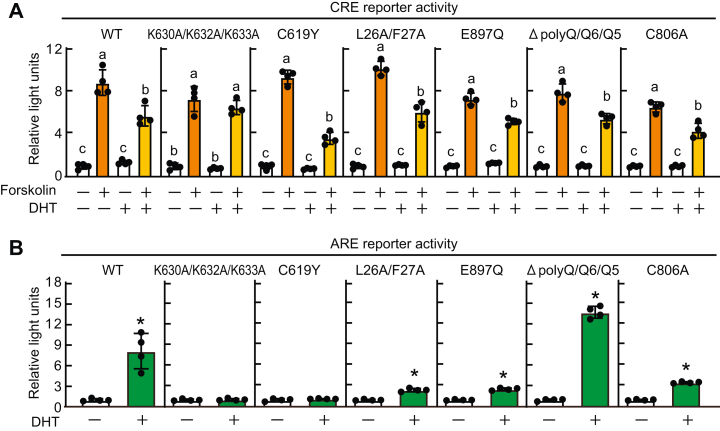
**Nuclear androgen receptor-suppressedCRE-mediated gene expression independent of its transcriptional activity.** HEK293FT cells transfected with androgen receptor expression vector pcDNA3.1-AR, pcDNA3.1-AR(K630A/K632A/K633A), pcDNA3.1-AR(C619Y), pcDNA3.1-AR(L26A/F27A), pcDNA3.1-AR(E897Q), pcDNA3.1-AR(ΔpolyQ+Q6+Q5), or pcDNA3.1-AR(C806A), luciferase reporter vector (*A*) p4xCRE-TATA-Luc2P or (*B*) pGL4-ARE2-TATA-Luc, and *Renilla* luciferase reporter vector pGL4.74[*hRluc*/TK] for 24 h. *A*, cells were incubated in the presence of 10 nM DHT for 16 h and then stimulated with 10 μM forskolin for an additional 4 h. *B*, cells were incubated in the presence of 10 nM DHT for 24 h. Luciferase reporter assays were performed. Data are expressed as means ± SD. (n = 4). Different letters or *asterisks* indicate significant differences (*p* < 0.05). ARE, androgen receptor response element; CRE, cAMP response element; DHT, dihydrotestosterone.

We used AR mutants to further investigate the mechanism by which AR suppressed CRE activation. As shown in Figure 6*A*, a K630A/K632A/K633A mutant of AR, which does not enter the nucleus owing to disruption of the nuclear localization signal (31), was unable to suppress CRE activity. In contrast, a C619Y mutant, which lacks the ability of AR to bind to DNA (32), retained its repressive activity. Unlike wild-type AR, both the K630A/K632A/K633A and C619Y mutants lacked transcriptional activity (Fig. 6*B*). Other AR mutants, such as L26A/F27A or E897Q, which both lack the N-terminal and C-terminal interaction capacity of AR (33, 34); ΔpolyQ+Q6+Q5, which lacks three glutamine tracts (35); and C806A, which lacks the palmitoylation site for cellular membrane translocation (36) had no effect on the ability of AR to repress CRE activity (Fig. 6*A*). Collectively, these results indicate that AR nuclear localization, but not transcriptional activity, is required for the AR-mediated repression of CRE.

### AR formed a protein complex with CREB

**Figure 7:**
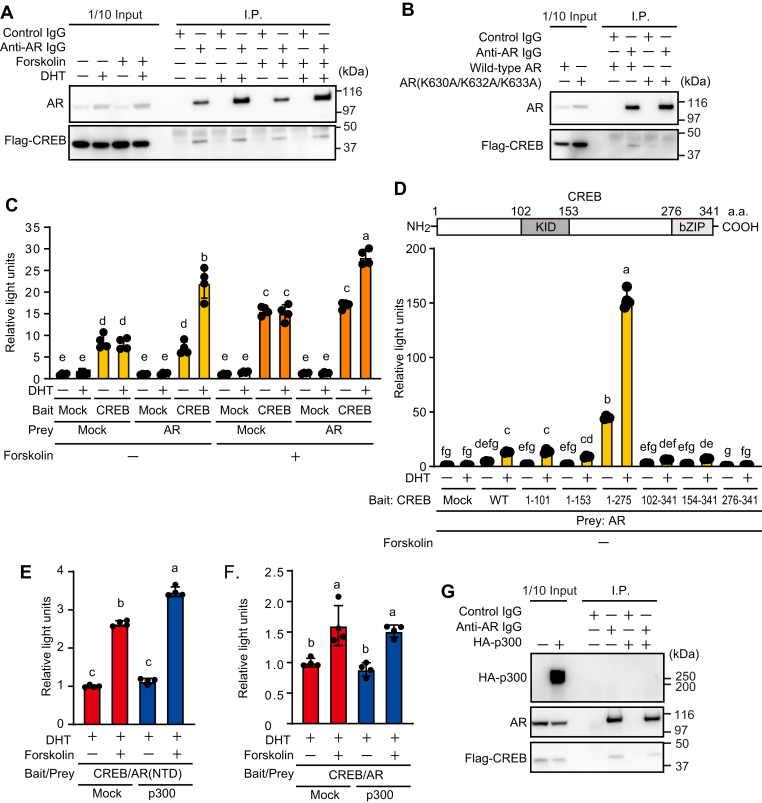
**AR bound to CREB in a modified mammalian two-hybrid assay *in vitro*.***A*, *B*, and *G*, HEK293FT cells were transiently transfected with pcDNA3.1-AR or pcDNA3.1-AR(K630A/K632A/K633A), pCF CREB, and pSG5 or pSG5-HA-p300. The cells were treated with 10 nM DHT for 16 h and further incubated in the presence of 10 μM forskolin for 5 min. The cells were harvested, and immunoprecipitation was performed. Proteins bound to the resin were analyzed by Western blotting. The graphs are a representative of three independent experiments. *C* and *D*, HEK293FT cells were transiently transfected with pBIND or pGAL-CREB, pcDNA3.1 or pcDNA3.1-AR, pG5luc, and pGL4.74[*hRluc*/TK] for 24 h. *E* and *F*, HEK293FT cells were transiently transfected with pGAL-CREB, pACT-AR(NTD) or pcDNA3.1-AR, pG5luc, pGL4.74[*hRluc*/TK], and pSG5 (mock) or pSG5-HA-p300. pACT-AR(NTD) or pcDNA3.1-AR to pSG5-HA-p300 ratio was adjusted to 1:2.5. The cells were treated with 10 nM DHT for 16 h and further incubated in the presence of 10 μM forskolin for 4 h. Luciferase activity is expressed in relative light units. Data are expressed as mean ± SD. (n = 4). Different letters indicate statistical differences (*p* < 0.05). AR, androgen receptor; CREB, cAMP-response element-binding protein; DHT, dihydrotestosterone; NTD, N-terminal domain.

We examined the protein interactions between overexpressed AR and CREB using immunoprecipitation in HEK293FT cells. Flag-CREB was coimmunoprecipitated with both ligand-bound and ligand-unbound AR, irrespective of forskolin stimulation (Fig. 7*A*). In contrast, Flag-CREB was not coimmunoprecipitated with a mutant AR (K630A/K632A/K633A) lacking nuclear localization ability (Fig. 7*B*). This suggests that the nuclear AR interacts with CREB. We further assessed the ability of AR to interact with CREB using a modified mammalian two-hybrid assay in which a nuclear receptor ligand dependently transactivates a luciferase reporter gene when the receptor binds to a bait protein. This assay allows the evaluation of binding between a nuclear receptor (*i.e.*, AR) and a bait protein (*i.e.*, CREB) only in the presence of an agonist for a nuclear receptor (*i.e.*, DHT). When GAL4DBD-fused CREB was expressed, the luciferase reporter was induced, presumably by the transcriptional activity of CREB. This is supported by the finding that reporter gene activity was significantly activated in the presence of forskolin. Moreover, the ability of GAL4DBD-fused CREB to stimulate reporter gene activity was further enhanced by AR in a DHT-dependent manner (Fig. 7*C*). The binding site of CREB that interacts with AR was determined using a modified mammalian two-hybrid assay. Luciferase reporter activity was significantly enhanced by AR activation when CREB mutants containing the N-terminal region (amino acids 1–101) were used as the bait protein (Fig. 7*D*). Although AR activation also enhanced luciferase activity when CREB mutants not containing the 1 to 101 region were used as the bait protein (Student’s *t* test: 102–341, *p* < 0.05; 154–341, *p* < 0.05; 276–341, and *p* < 0.05), they did not reach statistical significance in multiple comparisons. These results suggest that the N-terminal region of CREB is effective but that multiple regions of CREB may be involved in binding to the AR. It was previously shown that the N-terminal domain (NTD) of AR can form a protein complex with CREB bridged by p300 (27). We successfully reproduced this result with the mammalian two-hybrid assay (Fig. 7*E*). In contrast, p300 had no effect on the complex formation when the NTD of AR was replaced with full-length AR (Fig. 7*F*). An immunoprecipitation assay supported the notion that the binding of AR and CREB is not affected by p300 (Fig. 7*G*).

## Discussion

BAT is responsible for metabolic heat production, thereby controlling body temperature. Sex differences in body temperatures are observed in rodents and humans, with males exhibiting lower body temperature than in females (15, 16, 17). Accordingly, both castrated mice and ARKO mice exhibited higher body temperatures (22, 23). It has been proposed that AR suppresses *Ucp1* expression based on studies with primary and immortalized brown adipocytes (26, 37). Recent evidence suggests that BAT thermogenesis contributes to normal homeostatic thermoregulation under thermoneutral temperatures for breeding mice (9, 10). However, it has not been clarified whether androgens affect β-adrenoceptor-mediated thermogenesis *in vivo*. Further, the mechanistic roles of androgens in thermoregulation have not been elucidated. In the present study, we showed that AR-mediated androgen signaling suppressed the thermogenesis elicited by the β-adrenergic agonist *via* the repression of CREB phosphorylation and *Ucp1* expression *in vivo*, presumably by acting on BAT. In addition, activated AR suppressed CRE-mediated gene expression by forming a protein complex with CREB and repressing CREB phosphorylation. Taken together, these results indicate that androgen-activated AR suppresses β3-AdR/CREB/CRE/UCP1-mediated thermogenesis in BAT by forming a protein complex with CREB and suppressing its phosphorylation.

Male control mice showed an age-related decrease in body temperature, and differences in body temperature between control and ARKO mice were observed from 8 weeks of age. This time frame coincides with an alteration of testosterone levels, which rise from 4 weeks of age and lead to sexual maturation at approximately 8 weeks of age (11, 12). In contrast, mice castrated at 8 weeks of age had a higher body temperature than sham-operated mice after 2 weeks of the operation. Similar to the normal body temperature increases in both castrated and ARKO male mice (23, 24, 25), these mice also showed β3-agonist-induced increases in their body temperature, strongly suggesting that the thermogenesis caused by the deficiency of androgen action is regulated by a common mechanism. Although recent studies proposed that TRPM8 (38), OXER1 (39), GPRC6A (40), and ZIP9 (41) act as membrane ARs (42), the present study provides evidence that androgen–AR signaling contributes to lower body temperature.

Similar to castrated male mice (43), serum testosterone and DHT levels are markedly decreased in global ARKO male mice (44), which may be owing to their atrophic testes. As testosterone can be converted to 17β-estradiol by aromatase, our results from the *in vivo* study cannot exclude the possibility that 17β-estradiol is altered by castration or ARKO and is involved in the observed effects. However, a recent study using highly sensitive and specific GC/MS/MS shows that both intact and castrated male mice have extremely low circulating 17β-estradiol levels, as do ovariectomized female mice (43). In addition, 17β-estradiol stimulates BAT thermogenesis acting on the hypothalamus but not in peripheral tissues in female mice (45). CL316243 is selective to β3-AdR (28), which is highly expressed in white adipose tissue and BAT (46). Although β3-AdR is also expressed in the hypothalamus, hypothalamic β3-AdR is not involved in thermoregulation (47). Therefore, it is reasonable to expect that androgens, but not estrogen, are involved in the observed β3-agonist-induced thermogenesis in the castrated and ARKO mice.

The increase of body temperature by the β-adrenergic agonist was greater in ARKO and castrated mice than in their respective controls, whereas the expression levels of BAT *β3-AdR* was similar in control and ARKO mice (Fig. 4*C*). The latter was supported by the evidence that BAT β3-AdR protein levels do not differ between young adult male and female young adult rats (48). It is therefore suggested that BAT β3-AdR expression is not affected by sex or male hormones. Similarly, the induction of *Ucp1* by the β-adrenergic agonist was higher in ARKO mice, whereas the *Pgc1α* induction was similar between control and ARKO mice. This difference is explained by the fact that the expression of *Ucp1* and *Pgc1α* is differentially regulated by PKA (8). PKA/p38MAPK/ATF2 increases both the expression of *Ucp1* and *Pgc1α*, whereas PKA/CREB increases *Ucp1* alone (8). Our results that CREB activation was enhanced by ARKO supports the notion that *Pgc1α* expression is independent of CREB activation.

Cold exposure affects noradrenaline release from sympathetic nerves within the hypothalamus (2) and is associated with sexual differences in UCP1 expression in the BAT of rats (19). Both the release of noradrenaline from sympathetic nerves innervating BAT in rats (49) and blood noradrenaline levels in humans (50) respond to cold exposure more strongly in males than in females, despite the fact that estrogen receptor α induces noradrenaline release from the hypothalamus leading to thermogenesis in BAT (51). Taking AR expression in the hypothalamus (52) into account, androgens can also affect noradrenaline release. To eliminate the influence of endogenous noradrenaline, we employed β-adrenergic agonist treatment instead of cold exposure to determine the role of androgen signaling in BAT thermogenesis, and our results were similar to a recent study that demonstrates that UCP1 expression levels are higher in the WAT of castrated rats than sham-operated mice under cold exposure (53). Our study reveals the direct effects of androgen–AR signaling on the β3-AdR signaling in BAT.

AR inhibited CRE-mediated transcription by suppressing CREB phosphorylation, presumably by forming a protein complex with CREB. This action of AR was independent of its transcriptional activity but required AR nuclear localization. This result would lead to an outcome opposite to that of a pharmacological dose of testosterone, which can inhibit PDE activity and increase cAMP levels (54). In addition to PKA signaling, CREB can be phosphorylated and activated by PKC and MAPK signaling. Activation of AR stimulates the phosphorylation of CREB (Ser133) in a PKC-dependent manner in hippocampal neurons (55) and in an ERK-dependent manner in prostate cancer cells (56) and Sertoli cells (57). In these studies, the effects of AR on CREB differ from those in this study. In spermatogenic cells (58), testosterone induced the phosphorylation of CREB (Ser133) in an AR-independent manner (58), suggesting a role played by other ARs (*e.g.*, TRPM8 (38), OXER1 (39), GPRC6A (40), or ZIP9 (41)). Collectively, these results suggest that AR selectively inhibits CREB phosphorylation by PKA. In contrast, PKA signaling enhances the transcription of AR in prostate cancer cells (27). The NTD of AR and CREB can form a protein complex bridged by p300 (27), which was reproduced in this study. However, in our modified mammalian two-hybrid system, p300 did not form a protein complex with full-length AR. This discrepancy may result from structural differences between the forms of AR (*i.e.*, the NTD of AR and full-length AR). These results suggest that full-length AR can form protein complex with CREB even in the absence of p300.

Meta-analyses of epidemiological studies indicate that a low testosterone level increases the risks of cardiovascular and all-cause death (59, 60), although not all experiments support this notion (61, 62). Several studies, including a large longitudinal cohort analysis, show that a high core body temperature is associated with a short lifespan (17, 63, 64). High-fat diet-fed ARKO mice also exhibited shortened lifespans, accompanying high core body temperatures (22). Therefore, the mechanisms we found in this study may contribute to early death *via* hyperthermia.

In this study, we show that androgen reduces adrenaline-induced thermogenesis by suppressing adrenaline-activated CREB phosphorylation in BAT. A limitation of this study is that it was performed in mice, and further studies are needed to determine if the current results observed in mice are consistent in humans, as some differences are seen between mouse and human BAT. Since CREB has various physiological roles (65), crosstalk between AR and CREB is expected to be involved in a variety of physiological and pathophysiological processes. Future studies are warranted to further understand the importance of this interaction in biological processes.

## Experimental procedures

### Animals

Male global ARKO mice (CAG-*cre*; *Ar*^*Δ/y*^) were generated using the Cre-loxP system as described previously (22). Briefly, female AR^*flox/flox*^ mice (66) (C57BL/6 background) were crossed with CAG*-cre* mice (67) (C57BL/6 background) in which the expression of Cre recombinase is controlled by a ubiquitous CAG promoter. Male *Ar*^*flox/y*^ littermates were used for their controls. The mice were weaned at 4 weeks of age. For the castration studies, male 7-week-old C57BL/6J mice were obtained from Kiwa Laboratory Animals Co and castrated or sham operated at 8 weeks of age under anesthesia. Sham-operated mice and castrated mice were separated so that there was no difference in core body temperature before the operation. Mice were maintained under controlled temperatures (23 °C ± 3 °C) and a 12:12-h light: dark cycle (lighting period starting at 08:00) with *ad libitum* access to standard chow food (CE2, CLEA Japan Inc) and water. Rectal temperature was measured with a thermometer (KN-91, Natsume Seisakusho Co, Ltd) between 14:00 and 15:00 to evaluate weekly changes. At 12 weeks of age, sham-operated and castrated mice were intraperitoneally injected with vehicle (4% DMSO in saline) between 13:30 and 14:30, and body temperature was analyzed for 3 h. The next day, the vehicle was changed to 0.1 mg/kg CL316243 and body temperature was again measured for 3 h. Mice were euthanized at 13 weeks of age under anesthesia 2 h after injection with 0.1 mg/kg CL316243 (between 13:30 and 14:30), and the plasma and organs were obtained for further analysis. Free fatty acid level in plasma was determined using the NEFA C-Test (FUJIFILM Wako Pure Chemical Industries, Ltd). Mice from the two groups were alternately treated and dissected. All animal experiments were approved by the Animal Care and Use Committee of Osaka Prefecture University (Nos. 20-29, 21-21, and 21-22) and were performed in compliance with its guidelines.

### Cell culture

Mouse brown adipocyte T37i cells (Sigma-Aldrich) or HEK293FT cells (Thermo Fisher Scientific) were cultured in Dulbecco's Modified Eagle Medium/Nutrient Mixture F-12 Ham (1:1 Mixture) or Dulbecco's Modified Eagle Medium supplemented with 10% fetal bovine serum, 100 units/ml penicillin, and 100 mg/ml streptomycin. Steroid-free medium was prepared using dextran-coated charcoal-treated 10% fetal bovine serum and phenol red-free medium and used for all experiments for the determination of ligand dependency. Cells were maintained at 37 °C in an atmosphere containing 5% CO_2_ and 95% air atmosphere.

### Plasmids

Mutant AR expression vectors pcDNA3.1-AR(K630A/K632A/K633A) and pcDNA3.1-AR(C806A), in which Ala was substituted for Lys630/Lys632/Lys633 or Cys806, respectively, were constructed by site-directed mutagenesis using specific primers (sense: 5′-CTCTGGGAGCCCGGGCACTGGCAGCACTTGGTAATCTGAAAC-3′ and antisense: 5′-GTTTCAGATTACCAAGTGCTGCCAGTGCCCGGGCTCCCAGAG-3′ or sense: 5′-CCCAGGAATTCCTGGGCATGAAAGCACTGC-3′ and antisense: 5′-GCAGTGCTTTCATGCCCAGGAATTCCTGGG-3′) and pcDNA3.1-AR (68) as a template. The Flag-tagged CREB expression vector pCF CREB, the GAL4BD-fused CREB expression vector pGAL-CREB, and pSG5-HA-p300 were kindly provided by Dr Marc Montminy (Addgene, #22968) (69), Dr Ugo Moens (Addgene, #15221) (70), and Dr Elizabeth Wilson (Addgene, #89094) (71), respectively. For construction of pUCP1-pro-Luc2P, the 3.8 kbp of mouse *Ucp1* promoter and TK promoter from pUCP1-pro-Luc (72) were inserted into pGL4.11 (Promega). The CRE2 (73) and CRE4 (8) mutants of pUCP1-pro-Luc2P were constructed by site-directed mutagenesis using specific primers (sense: 5′-ACCACACTGAACTAGTTGTCACCTTTCCACTC-3′ and antisense: 5′-GAGTGGAAAGGTGACAACTAGTTCAGTGTGGT-3′ or 5′-AGGGCTTTGGGAGTGTGGCGCGGCTGGGAG-3′ and 5′-CTCCCAGCCGCGCCACACTCCCAAAGCCCT-3′).

### Luciferase reporter assay and modified mammalian two-hybrid assay

HEK293FT cells were seeded in 48-well plates at 6.0 × 10^4^ cells/well using steroid-free medium and cultured overnight. For determination of CRE- or androren response element (ARE)-mediated transcriptional activity, cells were transfected with β3-AdR expression vector (pcDNA3.2-β3-AdR-V5 (74)), AR expression vector (pcDNA3.1-AR (68), pcDNA3.1-AR (K630A/K632A/K633A), pcDNA3.1-AR (C619Y) (32, 75), pcDNA3.1-AR (L26A/F27A) (76), pcDNA3.1-AR (E897Q) (76), pcDNA3.1-AR (ΔpolyQ+Q6+Q5) (35), pcDNA3.1-AR (C806A)), luciferase reporter vector (p4xCRE-TATA-Luc2P (74), pGL4-ARE2-TATA-Luc (77), or pUCP1-pro-Luc2P), and *Renilla* luciferase reporter vector (pGL4.74[*hRluc*/TK] (Promega)) with PEI MAX (Polysciences Inc) and Opti-MEM (Thermo Fisher Scientific) for 24 h. For the mammalian two-hybrid assay, cells were transfected with pGAL-CREB, pcDNA3.1-AR or pACT-AR (NTD) (76), pG5luc (Promega), and pGL4.74[*hRluc*/TK] with PEI MAX and Opti-MEM for 24 h. Cells were incubated in the presence of 10 nM DHT for 16 h and then stimulated with 10 μM CL316243 or 10 μM forskolin for an additional 4 h. Cells were treated with the AR antagonist bicalutamide (10 μM) 30 min prior to DHT treatment. Luciferase reporter activity was determined as described previously (68). Data are expressed as relative light units.

### Western blotting

BAT was homogenized in RIPA buffer containing phosphatase and protease inhibitors (50 mM Tris-HCl, pH7.5, 150 mM NaCl, 1% TritonX-100, 1% sodium deoxycholate, 0.1% SDS, 2 mM EDTA, 10 mM sodium pyrophosphate, 10 mM sodium molybdate, 10 mM sodium fluoride, 1 mM sodium orthovanadate, 10 μg/ml leupeptin, 1 μg/ml aprotinin, and 1 mM PMSF). HEK293FT cells were plated in steroid-free medium and transiently transfected with pcDNA3.1-AR for 24 h. Cells were cultured in the presence of 10 nM DHT for 16 h, followed by stimulation with 10 μM forskolin for 5 min. Cells were washed twice with TBS and harvested and lysed in buffer containing phosphatase and protease inhibitors (50 mM Tris-HCl, pH7.5, 150 mM NaCl, 0.5% NP-40, 2 mM EDTA, 10 mM sodium pyrophosphate, 10 mM sodium molybdate, 10 mM sodium fluoride, 1 mM sodium orthovanadate, 10 μg/ml leupeptin, 1 μg/ml aprotinin, and 1 mM PMSF). After sonication and centrifugation of the tissue and cell culture lysates, the supernatant was subjected to SDS-PAGE, followed by Western blotting with monoclonal anti-CREB (1:5000, 48H2, Cell Signaling Technology, RRID:AB_10699020), monoclonal anti-pCREB (10E9, Santa Cruz Biotechnology, RRID:AB_1125727), polyclonal anti-AR (N-20, 1:3000, Santa Cruz Biotechnology, RRID: AB_1563391 or RB3838 (antigen: KLH-conjugated the N-terminal 20 amino acids of AR, MEVQLGLGRVYPRPPSKTYR–Cys), 1/3000, GL Biochem), and monoclonal anti-β-actin (1:5000, 2D4H5, Proteintech, RRID:AB_2687938) antibodies. After immunoreaction with horseradish peroxidase-conjugated secondary antibodies (1:10,000, Bio-Rad), immunoreactive bands were developed using Immobilon Western Chemiluminescent HRP Substrate (Merck Millipore) and detected using an LAS4000 imager (GE Healthcare). Densitometry measurements were performed by using ImageJ software [version 1.4.3.67, RRID:SCR_003070, National Institutes of Health, https://imagej.nih.gov/].

### Immunoprecipitation

HEK293FT cells were plated in steroid-free medium and transiently transfected with pcDNA3.1-AR or pcDNA3.1-AR (K630A/K632A/K633A), pCF CREB, and pSG5 or pSG5-HA-p300 with PEI MAX for 24 h. Cells were cultured in the presence of 10 nM DHT for 16 h, followed by stimulation with 10 μM forskolin for 5 min. Cells were washed twice with PBS and harvested and lysed in buffer (20 mM Hepes-NaOH, pH7.5, 150 mM NaCl, 0.5% NP-40 substitute, 2 mM EDTA, 10% glycerol, 1 mM DTT, 10 μg/ml leupeptin, 1 μg/ml aprotinin, and 1 mM PMSF) on ice for 30 min. After centrifugation at 20,000*g* for 20 min, the supernatant (1 mg) was incubated with 1.5 μg of normal rabbit IgG (FUJIFILM Wako) or affinity purified anti-AR RB3838 IgG overnight at 4 °C, followed by reaction with Dynabeads Protein G (Veritas) for 2 h. The resin was washed four times with the same buffer, and proteins bound to the resin were analyzed by Western blotting.

### Preparation of complementary DNA and real-time PCR

Total RNA was isolated from mouse tissues using Sepasol RNA I super G (Nacalai Tesque) by disrupting cells with zirconia beads using a Multi-Beads Shocker (Yasui Kikai Corp), followed by treatment with DNase I. Complementary DNA was synthesized from total RNA using ReverTra Ace (TOYOBO), oligo(dT)20 primer, and random hexamer primers. Real-time PCR was performed using TB Green Premix *Ex Taq* II DNA polymerase (Takara Bio) and Thermal Cycler Dice TP870 (Takara Bio). The sequences of the specific primers for target genes and their annealing temperatures are described in Table S1. The relative expression of target genes was calculated by the standard curve method using Ct values and normalized by that of the control gene. The specificity of each PCR product was confirmed by electrophoresis and dissociation curve analysis.

### Statistical analysis

Data were analyzed by Student’s *t* test or one-way ANOVA with Tukey-Kramer’s or Dunnett’s *post hoc* tests using JMP statistical software (version 8.0.1., SAS Institute). Data are shown as means ± SD, and a *p*-value (*p*) *<* 0.05 was considered statistically significant. Significance is indicated in the figures by asterisks or different letters. Bars not sharing the same letter show significant differences between the groups.

## Data availability

Data are available within the article or its supplementary materials.

## Supporting information

This article contains supporting information.

## Conflict of interest

The authors declare that they have no conflicts of interest with the contents of this article.

## References

[1] Morrison S.F., Nakamura K. (2019). Central mechanisms for thermoregulation. Ann. Rev. Physiol..

[2] Cannon B., Nedergaard J. (2004). Brown adipose tissue: function and physiological significance. Physiol. Rev..

[3] van Marken Lichtenbelt W.D., Schrauwen P. (2011). Implications of nonshivering thermogenesis for energy balance regulation in humans. Am. J. Physiol. Regul. Integr. Comp. Physiol..

[4] Kajimura S., Saito M. (2014). A new era in brown adipose tissue biology: molecular control of brown fat development and energy homeostasis. Ann. Rev. Physiol..

[5] Virtanen K.A., Lidell M.E., Orava J., Heglind M., Westergren R., Niemi T. (2009). Functional brown adipose tissue in healthy adults. New Engl. J. Med..

[6] van Marken Lichtenbelt W.D., Vanhommerig J.W., Smulders N.M., Drossaerts J.M., Kemerink G.J., Bouvy N.D. (2009). Cold-activated brown adipose tissue in healthy men. New Engl. J. Med..

[7] Saito M., Okamatsu-Ogura Y., Matsushita M., Watanabe K., Yoneshiro T., Nio-Kobayashi J. (2009). High incidence of metabolically active brown adipose tissue in healthy adult humans: effects of cold exposure and adiposity. Diabetes.

[8] Cao W., Daniel K.W., Robidoux J., Puigserver P., Medvedev A.V., Bai X. (2004). p38 mitogen-activated protein kinase is the central regulator of cyclic AMP-dependent transcription of the brown fat uncoupling protein 1 gene. Mol. Cell. Biol..

[9] Schlein C., Fischer A.W., Sass F., Worthmann A., Tödter K., Jaeckstein M.Y. (2021). Endogenous fatty acid synthesis drives brown adipose tissue involution. Cell Rep..

[10] Feldmann H.M., Golozoubova V., Cannon B., Nedergaard J. (2009). UCP1 ablation induces obesity and abolishes diet-induced thermogenesis in mice exempt from thermal stress by living at thermoneutrality. Cell Metab..

[11] O'Shaughnessy P.J., Baker P.J., Johnston H. (2006). The foetal Leydig cell-- differentiation, function and regulation. Int. J. Androl..

[12] Wu X., Arumugam R., Zhang N., Lee M.M. (2010). Androgen profiles during pubertal Leydig cell development in mice. Reproduction.

[13] Romeo R.D. (2003). Puberty: a period of both organizational and activational effects of steroid hormones on neurobehavioural development. J. Neuroendocrinol..

[14] Hau M. (2007). Regulation of male traits by testosterone: implications for the evolution of vertebrate life histories. BioEssays.

[15] Sanchez-Alavez M., Alboni S., Conti B. (2011). Sex- and age-specific differences in core body temperature of C57Bl/6 mice. Age.

[16] Hoffmann M.E., Rodriguez S.M., Zeiss D.M., Wachsberg K.N., Kushner R.F., Landsberg L. (2012). 24-h core temperature in obese and lean men and women. Obesity.

[17] Obermeyer Z., Samra J.K., Mullainathan S. (2017). Individual differences in normal body temperature: longitudinal big data analysis of patient records. BMJ.

[18] Kaikaew K., Grefhorst A., Visser J.A. (2021). Sex differences in Brown adipose tissue function: sex hormones, glucocorticoids, and their crosstalk. Front. Endocrinol..

[19] Quevedo S., Roca P., Picó C., Palou A. (1998). Sex-associated differences in cold-induced UCP1 synthesis in rodent brown adipose tissue. Pflug. Arch. Eur. J. Physiol..

[20] Charkoudian N., Stachenfeld N.S. (2014). Reproductive hormone influences on thermoregulation in women. Compr. Physiol..

[21] van Veen J.E., Kammel L.G., Bunda P.C., Shum M., Reid M.S., Massa M.G. (2020). Hypothalamic estrogen receptor alpha establishes a sexually dimorphic regulatory node of energy expenditure. Nat. Metab..

[22] Harada N., Hanada K., Minami Y., Kitakaze T., Ogata Y., Tokumoto H. (2020). Role of gut microbiota in sex- and diet-dependent metabolic disorders that lead to early mortality of androgen receptor-deficient male mice. Am. J. Physiol. Endocrinol. Metab..

[23] Harada N., Hanaoka R., Hanada K., Izawa T., Inui H., Yamaji R. (2016). Hypogonadism alters cecal and fecal microbiota in male mice. Gut Microbes.

[24] Hashimoto O., Noda T., Morita A., Morita M., Ohtsuki H., Sugiyama M. (2016). Castration induced browning in subcutaneous white adipose tissue in male mice. Biochem. Biophys. Res. Commun..

[25] Clarke S.D., Clarke I.J., Rao A., Cowley M.A., Henry B.A. (2012). Sex differences in the metabolic effects of testosterone in sheep. Endocrinology.

[26] Rodriguez A.M., Monjo M., Roca P., Palou A. (2002). Opposite actions of testosterone and progesterone on UCP1 mRNA expression in cultured brown adipocytes. Cell. Mol. Life Sci..

[27] Kim J., Jia L., Stallcup M.R., Coetzee G.A. (2005). The role of protein kinase A pathway and cAMP responsive element-binding protein in androgen receptor-mediated transcription at the prostate-specific antigen locus. J. Mol. Endocrinol..

[28] Susulic V.S., Frederich R.C., Lawitts J., Tozzo E., Kahn B.B., Harper M.E. (1995). Targeted disruption of the beta 3-adrenergic receptor gene. J. Biol. Chem..

[29] Karamitri A., Shore A.M., Docherty K., Speakman J.R., Lomax M.A. (2009). Combinatorial transcription factor regulation of the cyclic AMP-response element on the Pgc-1alpha promoter in white 3T3-L1 and brown HIB-1B preadipocytes. J. Biol. Chem..

[30] Carpentier A.C., Blondin D.P., Virtanen K.A., Richard D., Haman F., Turcotte É E. (2018). Brown adipose tissue energy metabolism in humans. Front. Endocrinol..

[31] Cutress M.L., Whitaker H.C., Mills I.G., Stewart M., Neal D.E. (2008). Structural basis for the nuclear import of the human androgen receptor. J. Cell. Sci..

[32] Nazareth L.V., Stenoien D.L., Bingman W.E., James A.J., Wu C., Zhang Y. (1999). A C619Y mutation in the human androgen receptor causes inactivation and mislocalization of the receptor with concomitant sequestration of SRC-1 (steroid receptor coactivator 1). Mol. Endocrinol..

[33] He B., Gampe R.T.J., Kole A.J., Hnat A.T., Stanley T.B., An G. (2004). Structural basis for androgen receptor interdomain and coactivator interactions suggests a transition in nuclear receptor activation function dominance. Mol. Cell..

[34] Schaufele F., Carbonell X., Guerbadot M., Borngraeber S., Chapman M.S., Ma A.A. (2005). The structural basis of androgen receptor activation: intramolecular and intermolecular amino-carboxy interactions. Proc. Natl. Acad. Sci. U. S. A..

[35] Harada N., Mitani T., Higashimura Y., Yamaji R., Okamoto K., Nakano Y. (2010). Involvement of three glutamine tracts in human androgen receptor transactivation. J. Steroid Biochem. Mol. Biol..

[36] Pedram A., Razandi M., Sainson R.C., Kim J.K., Hughes C.C., Levin E.R. (2007). A conserved mechanism for steroid receptor translocation to the plasma membrane. J. Biol. Chem..

[37] Lerner A., Kewada D., Ahmed A., Hardy K., Christian M., Franks S. (2020). Androgen reduces mitochondrial respiration in mouse brown adipocytes: a model for disordered energy balance in polycystic ovary syndrome. Int. J. Mol. Sci..

[38] Asuthkar S., Elustondo P.A., Demirkhanyan L., Sun X., Baskaran P., Velpula K.K. (2015). The TRPM8 protein is a testosterone receptor: i. Biochemical evidence for direct TRPM8-testosterone interactions. J. Biol. Chem..

[39] Kalyvianaki K., Gebhart V., Peroulis N., Panagiotopoulou C., Kiagiadaki F., Pediaditakis I. (2017). Antagonizing effects of membrane-acting androgens on the eicosanoid receptor OXER1 in prostate cancer. Sci. Rep..

[40] Pi M., Parrill A.L., Quarles L.D. (2010). GPRC6A mediates the non-genomic effects of steroids. J. Biol. Chem..

[41] Thomas P., Converse A., Berg H.A. (2018). ZIP9, a novel membrane androgen receptor and zinc transporter protein. Gen. Comp. Endocrinol..

[42] Thomas P. (2019). Membrane androgen receptors unrelated to nuclear steroid receptors. Endocrinology.

[43] Nilsson M.E., Vandenput L., Tivesten A., Norlen A.K., Lagerquist M.K., Windahl S.H. (2015). Measurement of a comprehensive sex steroid profile in rodent serum by high-sensitive gas chromatography-tandem mass spectrometry. Endocrinology.

[44] Kawano H., Sato T., Yamada T., Matsumoto T., Sekine K., Watanabe T. (2003). Suppressive function of androgen receptor in bone resorption. Proc. Natl. Acad. Sci. U. S. A..

[45] Zhang Z., DiVittorio J.R., Joseph A.M., Correa S.M. (2021). The effects of estrogens on neural circuits that control temperature. Endocrinology.

[46] Krief S., Lönnqvist F., Raimbault S., Baude B., Van Spronsen A., Arner P. (1993). Tissue distribution of beta 3-adrenergic receptor mRNA in man. J. Clin. Invest..

[47] Richard J.E., López-Ferreras L., Chanclón B., Eerola K., Micallef P., Skibicka K.P. (2017). CNS β(3)-adrenergic receptor activation regulates feeding behavior, white fat browning, and body weight. Am. J. Physiol. Endocrinol. Metab..

[48] Rodriguez-Cuenca S., Pujol E., Justo R., Frontera M., Oliver J., Gianotti M. (2002). Sex-dependent thermogenesis, differences in mitochondrial morphology and function, and adrenergic response in brown adipose tissue. J. Biol. Chem..

[49] McDonald R.B., Hamilton J.S., Horwitz B.A. (1993). Influence of age and gender on brown adipose tissue norepinephrine turnover. Proc. Soc. Exp. Biol. Med..

[50] Solianik R., Skurvydas A., Vitkauskienė A., Brazaitis M. (2014). Gender-specific cold responses induce a similar body-cooling rate but different neuroendocrine and immune responses. Cryobiology.

[51] Martínez de Morentin P.B., González-García I., Martins L., Lage R., Fernández-Mallo D., Martínez-Sánchez N. (2014). Estradiol regulates brown adipose tissue thermogenesis *via* hypothalamic AMPK. Cell Metab..

[52] Dugger B.N., Morris J.A., Jordan C.L., Breedlove S.M. (2007). Androgen receptors are required for full masculinization of the ventromedial hypothalamus (VMH) in rats. Horm. Behav..

[53] Harnichar A.E., Zubiría M.G., Giordano A.P., Miguel I., Rey M.A., Spinedi E. (2022). Inhibitory effect of androgens on white adipose tissue thermogenic capacity. Mol. Cell. Endocrinol..

[54] Bordallo J., Cantabrana B., Suárez L., Sánchez M. (2011). Testosterone inhibits cAMP-phosphodiesterases in heart extracts from rats and increases cAMP levels in isolated left atria. Pharmacology.

[55] Nguyen T.V., Yao M., Pike C.J. (2009). Dihydrotestosterone activates CREB signaling in cultured hippocampal neurons. Brain Res..

[56] Unni E., Sun S., Nan B., McPhaul M.J., Cheskis B., Mancini M.A. (2004). Changes in androgen receptor nongenotropic signaling correlate with transition of LNCaP cells to androgen independence. Cancer Res..

[57] Fix C., Jordan C., Cano P., Walker W.H. (2004). Testosterone activates mitogen-activated protein kinase and the cAMP response element binding protein transcription factor in Sertoli cells. Proc. Natl. Acad. Sci. U. S. A..

[58] Shihan M., Bulldan A., Scheiner-Bobis G. (2014). Non-classical testosterone signaling is mediated by a G-protein-coupled receptor interacting with Gnα11. Biochim. Biophys. Acta Mol. Cell Res..

[59] Araujo A.B., Dixon J.M., Suarez E.A., Murad M.H., Guey L.T., Wittert G.A. (2011). Clinical review: endogenous testosterone and mortality in men: a systematic review and meta-analysis. J. Clin. Endocrinol. Metab..

[60] Corona G., Rastrelli G., Monami M., Guay A., Buvat J., Sforza A. (2011). Hypogonadism as a risk factor for cardiovascular mortality in men: a meta-analytic study. Eur. J. Endocrinol..

[61] Drori D., Folman Y. (1976). Environmental effects on longevity in the male rat: exercise, mating, castration and restricted feeding. Exp. Gerontol..

[62] Min K.J., Lee C.K., Park H.N. (2012). The lifespan of Korean eunuchs. Curr. Biol..

[63] Roth G.S., Lane M.A., Ingram D.K., Mattison J.A., Elahi D., Tobin J.D. (2002). Biomarkers of caloric restriction may predict longevity in humans. Science.

[64] Keil G., Cummings E., de Magalhães J.P. (2015). Being cool: how body temperature influences ageing and longevity. Biogerontology.

[65] Mayr B., Montminy M. (2001). Transcriptional regulation by the phosphorylation-dependent factor CREB. Nat. Rev. Mol. Cell Biol..

[66] Sato T., Matsumoto T., Kawano H., Watanabe T., Uematsu Y., Sekine K. (2004). Brain masculinization requires androgen receptor function. Proc. Natl. Acad. Sci. U. S. A..

[67] Matsumura H., Hasuwa H., Inoue N., Ikawa M., Okabe M. (2004). Lineage-specific cell disruption in living mice by Cre-mediated expression of diphtheria toxin A chain. Biochem. Biophys. Res. Commun..

[68] Harada N., Yasunaga R., Higashimura Y., Yamaji R., Fujimoto K., Moss J. (2007). Glyceraldehyde-3-phosphate dehydrogenase enhances transcriptional activity of androgen receptor in prostate cancer cells. J. Biol. Chem..

[69] Du K., Asahara H., Jhala U.S., Wagner B.L., Montminy M. (2000). Characterization of a CREB gain-of-function mutant with constitutive transcriptional activity *in vivo*. Mol. Cell. Biol..

[70] Delghandi M.P., Johannessen M., Moens U. (2005). The cAMP signalling pathway activates CREB through PKA, p38 and MSK1 in NIH 3T3 cells. Cell. Signal..

[71] Askew E.B., Bai S., Blackwelder A.J., Wilson E.M. (2010). Transcriptional synergy between melanoma antigen gene protein-A11 (MAGE-11) and p300 in androgen receptor signaling. J. Biol. Chem..

[72] Sakamoto T., Takahashi N., Sawaragi Y., Naknukool S., Yu R., Goto T. (2013). Inflammation induced by RAW macrophages suppresses UCP1 mRNA induction *via* ERK activation in 10T1/2 adipocytes. Am. J. Physiol. Cell Physiol..

[73] Yubero P., Barberá M.J., Alvarez R., Viñas O., Mampel T., Iglesias R. (1998). Dominant negative regulation by c-Jun of transcription of the uncoupling protein-1 gene through a proximal cAMP-regulatory element: a mechanism for repressing basal and norepinephrine-induced expression of the gene before brown adipocyte differentiation. Mol. Endocrinol..

[74] Harada N., Okuyama M., Teraoka Y., Arahori Y., Shinmori Y., Horiuchi H. (2022). Identification of G-protein coupled receptor 55 (GPR55) as a target of curcumin. Npj Sci. Food.

[75] Harada N., Katsuki T., Takahashi Y., Masuda T., Yoshinaga M., Adachi T. (2015). Androgen receptor silences thioredoxin-interacting protein and competitively inhibits glucocorticoid receptor-mediated apoptosis in pancreatic β-cells. J. Cell. Biochem..

[76] Harada N., Ohmori Y., Yamaji R., Higashimura Y., Okamoto K., Isohashi F. (2008). ARA24/Ran enhances the androgen-dependent NH2- and COOH-terminal interaction of the androgen receptor. Biochem. Biophys. Res. Commun..

[77] Harada N., Inoue K., Yamaji R., Nakano Y., Inui H. (2012). Androgen deprivation causes truncation of the C-terminal region of androgen receptor in human prostate cancer LNCaP cells. Cancer Sci..

