# Supplementary material for: Androgen receptor suppresses β-adrenoceptor-mediated CREB activation and thermogenesis in brown adipose tissue of male mice
Source: J Biol Chem. 2022 Oct 19;298(12):102619. doi: 10.1016/j.jbc.2022.102619 (PMC9700029; doi:10.1016/j.jbc.2022.102619)
Supplement: Table S1 [file mmc1.pdf]

Table S1. Primers for amplification of genes by qRT-PCR

Gene		Sequences of primers	X (°C)
Actb	Fw	5′-TTGCTGACAGGATGCAGAAG-3′	60
	Rv	5′-GTACTTGCGCTCAGGAGGAG-3′	
Ar	Fw	5′-GCAGCTTGTGCATGTGGTCA-3′	60
	Rv	5′-AATACCATCAGTCCCATCCAGGAA-3′	
Ucp1	Fw	5′-GTGAAGGTCAGAATGCAAGC-3′	58
	Rv	5′-AGGGCCCCCTTCATGAGGTC-3′	
Pgc1α	Fw	5′-AGCCGTGACCACTGACAACGAG-3′	58
	Rv	5′-GCTGCATGGTTCTGAGTGCTAAG-3′	
Adrb3	Fw	5′-AGAAACGGCTCTCTGGCTTTG-3′	55
	Rv	5′-TGGTTATGGTCTGTAGTCTCGG-3′	
Prdm16	Fw	5′-CAGCACGGTGAAGCCATTC-3′	55
	Rv	5′-GCGTGCATCCGCTTGTG-3′	
Cidea	Fw	5′-ATGGAGACCGCCAGGGACTAC-3′	60
	Rv	5′-GCTACTTCGGTCATGGTTTG-3′	
